# Targeting oxidative phosphorylation as an approach for the treatment of ovarian cancer

**DOI:** 10.3389/fonc.2022.971479

**Published:** 2022-09-06

**Authors:** Yinjie Wu, Xuewei Zhang, Ziyi Wang, Wanzhen Zheng, Huimin Cao, Wenjing Shen

**Affiliations:** ^1^ Department of Gynecology, The First Affiliated Hospital of China Medical University, Shenyang, China; ^2^ Department of Thoracic Surgery, The First Affiliated Hospital of China Medical University, Shenyang, China; ^3^ Department of Health Statistics, School of Public Health, China Medical University, Shenyang, China

**Keywords:** metabolic reprograming, oxidative phoshorylation, mitochondria, ovarian cancer, resistance

## Abstract

Ovarian cancer is an aggressive tumor that remains to be the most lethal gynecological malignancy in women. Metabolic adaptation is an emerging hallmark of tumors. It is important to exploit metabolic vulnerabilities of tumors as promising strategies to develop more effective anti-tumor regimens. Tumor cells reprogram the metabolic pathways to meet the bioenergetic, biosynthetic, and mitigate oxidative stress required for tumor cell proliferation and survival. Oxidative phosphorylation has been found to be altered in ovarian cancer, and oxidative phosphorylation is proposed as a therapeutic target for management of ovarian cancer. Herein, we initially introduced the overview of oxidative phosphorylation in cancer. Furthermore, we discussed the role of oxidative phosphorylation and chemotherapeutic resistance of ovarian cancer. The role of oxidative phosphorylation in other components of tumor microenvironment of ovarian cancer has also been discussed.

## Introduction

Ovarian cancer is an aggressive tumor that remains to be the most lethal gynecological malignancy in women (1). Resistance to conventional chemotherapeutic regimens is the leading cause of death for ovarian cancer patients. Recent studies have further investigated the biological behaviors of ovarian cancer cells and identified signaling pathways related to metabolic adaptation (2). Targeting these metabolism-related pathways represents a promising therapeutic strategy for overcoming chemotherapeutic resistance and reducing its recurrence rate in patients with ovarian cancer, whereas more efforts should be paid to raise our understanding of the underlying mechanisms of metabolic adaptation in ovarian cancer.

Dysregulated cellular energetics are emerging hallmarks of tumors. Tumor occurrence and development requires metabolic reprogramming of tumor cells. Tumor cells reprogram the metabolic pathways to meet the bioenergetic and biosynthetic demands required for tumor cell proliferation and survival (3). Additionally, the tumor microenvironment (TME) requires tumor cells capabilities to adapt to the nutrient-deprived and hypoxic environment to sustain tumor survival through diverse metabolic pathways (4, 5). Growing evidence has illustrated that metabolic phenotyping of components other than tumor cells within the TME, including immune cells, adipocytes, and cancer-associated fibroblasts, are also essential for tumor development (4).

## Overview of oxidative phosphorylation in cancer

**Figure 1:**
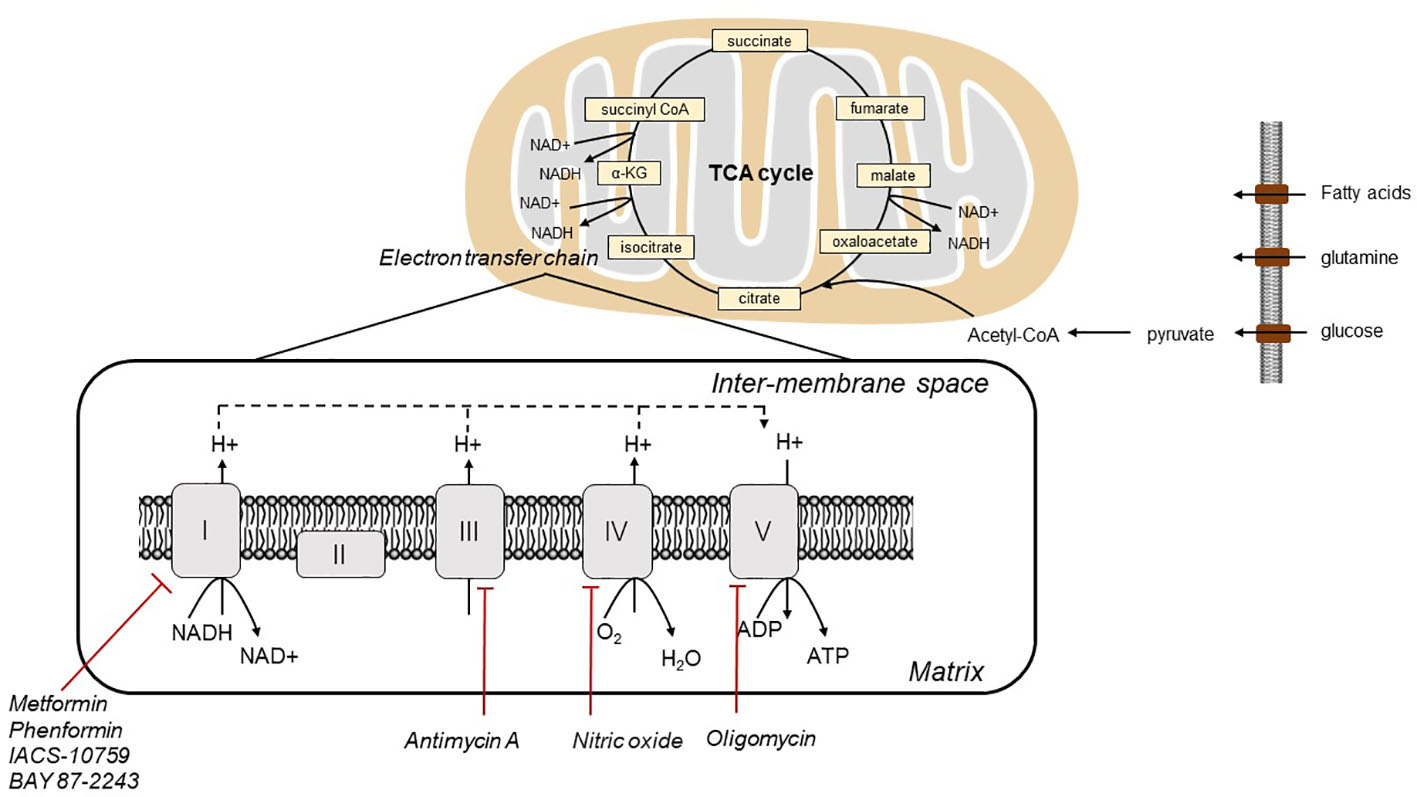
Illustration of mitochondrial electron transport, oxygen consumption and OXPHOS in cells.

In 1920s, Otto Warburg first discovered that tumor cells rely on glycolysis for ATP production, irrespective of the presence of oxygen. The contribution of oxidative phosphorylation (OXPHOS) in tumor cells has remained controversial. As electrons pass through the ETC *via* four mitochondrial protein complexes, namely NADH-Q oxidoreductase (complex I), succinate-Q reductase (complex II), Q-cytochrome c oxidoreductase (complex III), and cytochrome c oxidase (complex IV), protons are pumped from the mitochondrial matrix into the intermembrane space, which sets up the proton gradient. Complex V (ATP synthase) depends on the gradient to drive ATP generation *via via* (6). (**Figure 1**) Tumor cells have been reported to display enhanced aerobic glycolysis and impaired OXPHOS. In contrast to this traditional concept, although mutations of mitochondrial genes are commonly observed in tumor cells, mitochondrial energy metabolism is not inactivated, whereas the mitochondrial bioenergetic state is altered (7, 8). OXPHOS is active in ovarian tumor cells. It has been demonstrated that the reliance of ovarian tumor cells on OXPHOS is closely related to the survival and proliferation of cancer initiating stem cells. These surviving cancer stem cells had increased mitochondrial biogenesis with higher OXPHOS level (9). Thus, OXPHOS is proposed as a therapeutic target for management of ovarian cancer (9). Therefore, OXPHOS plays a key role in tumorigenesis of ovarian cancer, and targeting OXPHOS is a promising therapeutic strategy.

## Oxidative phosphorylation and chemotherapeutic resistance of ovarian cancer

**Figure 2:**
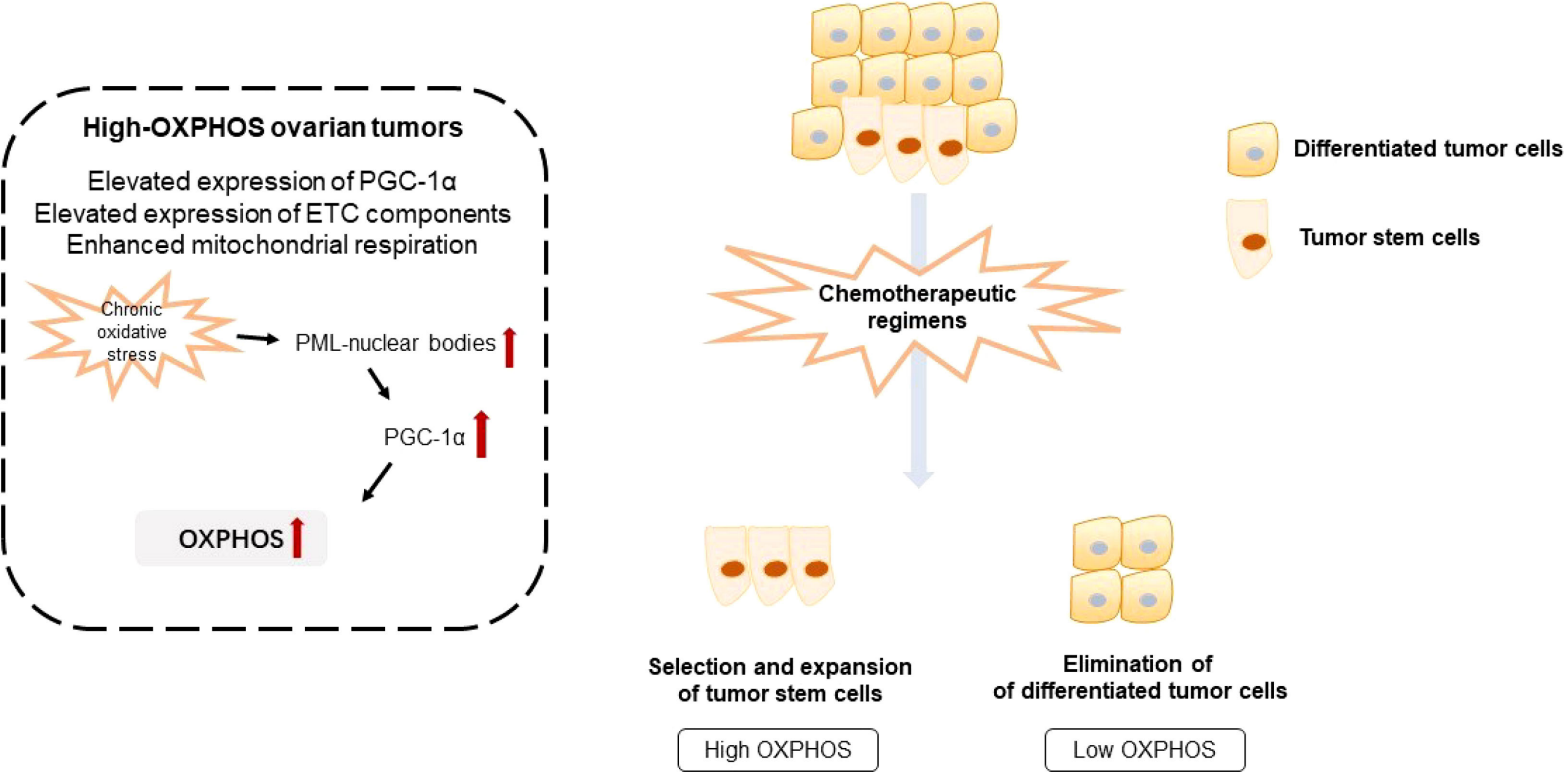
Illustration of oxidative phosphorylation and chemotherapeutic resistance of ovarian tumors. OXPHOS, oxidative phosphorylation; ETC, electron transport chain.

The conventional chemotherapeutic regimen for patients with ovarian cancer is a combination of paclitaxel and carboplatin, which selectively target and eliminate fast-proliferating tumor cells (10). In poorly vascularized and hypoxic regions of tumors, environmental factors endow tumor cells to be quiescent and unresponsive to chemotherapeutic regimens. Indeed, OXPHOS may not be limited by poor oxygen supply in hypoxic tumors, and ATP production from OXPHOS in tumors can be achieved at low oxygen concentration (7). OXPHOS inhibition could be an effective way to reduce the consumption of oxygen and to consequently increase oxygen availability in the tissue. As a result, oxygen could diffuse into initially hypoxic tumor regions, reducing tumor hypoxia (7). Furthermore, this could be a potential strategy for all hypoxic tumors, not simply those in which OXPHOS is upregulated. OXPHOS has been regarded as a critical metabolic vulnerability in chemotherapy-resistant tumors (11) (**Figure 2**).

Numerous studies have pointed out the existence of tumor subgroups with a preference for either aerobic glycolysis or OXPHOS (11). Combined proteomic, metabolomic, and bioenergetic analyses revealed that two heterogenous metabolic subgroups co-exist in ovarian tumors, namely low- and high-OXPHOS. High-OXPHOS tumors are characterized by elevated levels of electron transport chain components and enhanced mitochondrial respiration, while low-OXPHOS tumors exhibit a glycolytic phenotype. In high-OXPHOS tumors, chronic oxidative stress enhanced aggregation of PML-nuclear bodies, leading to activation of the transcriptional co-activator PGC-1α. Active PGC-1α further promotes the expression of electron transport chain complexes, thus increasing OXPHOS. Importantly, high-OXPHOS tumors display increased response to conventional chemotherapies, such as taxane and platinum (12). Therefore, metformin, respiratory complex I inhibitor, elevates mitochondrial ROS production and enhances cell death of high-OXPHOS cells, whereas it has little anti-tumor effect on low-OXPHOS cells.

Chemotherapeutic regimens promote selection and expansion of high-OXPHOS cancer stem cells. Combination of chemotherapeutic regimens with anti-tumor drugs targeting OXPHOS exerts a synergistic effect to improve the anti-tumor effect in ovarian cancer, indicating a promising therapeutic approach for chemotherapy-resistant ovarian tumors. Metabolic analysis revealed that resistant ovarian tumor cells undergo a metabolic shift towards OXPHOS. This metabolic shift coordinates with a re-organization of the mitochondrial network and accumulates mitochondrial components (13). Chemotherapy-resistant ovarian tumor cells display enhanced OXPHOS compared with the sensitive counterpart. After treatment with complex I inhibitor, metformin, and complex V inhibitor, oligomycin, cisplatin sensitivity is restored. Tumor necrosis factor-associated protein 1 (TRAP1), the mitochondrial isoform of heat shock protein 90, is a key regulator of metabolism (14). TRAP1 mediates a metabolic shift toward OXPHOS, which can trigger altered cytokines generation and gene expression within immune cells, ultimately resulting in cisplatin resistance and metastasis in ovarian cancer (15). Importantly, metabolic features of ovarian tumor cells have predictive value for cisplatin sensitivity (14). TRAP1, as a bioenergetic index and proinflammatory molecules, is a predictive and prognostic biomarker of chemotherapeutic outcome (16). Besides, as PGC1α is a key molecule for integrating and coordinating nuclear DNA and mitochondrial DNA transcriptional machinery, PGC1α may provide a target to improve chemotherapy efficacy. PGC1α mediates OXPHOS engaged in cisplatin resistance of ovarian tumor cells *via* nucleo-mitochondrial transcriptional feedback (17, 18). High expression of PGC1α confers on the tumor a unique molecular signature, resulting in elevated OXPHOS and mitochondrial biogenesis. Elevated OXPHOS ultimately conferred vulnerability to OXPHOS inhibition (19). NADH production in normal cells is reliant on the TCA cycle, while electron transport in tumor cells is highly reliant on cytosolic NADH produced by dehydrogenases, such as aldehyde dehydrogenase (ALDH) (20, 21). Targeting OXPHOS in tumor cells by inhibiting ALDH to reduce NADH generation can selectively reduce ATP production, suppressing autophagy and causing tumor cell death (22).

## Oxidative phosphorylation and TME of ovarian cancer

Considering that diverse and dynamic interactions of tumor cells and other components of TME may exert a profound effect on the metabolic adaptation of tumor, immune and stromal cells, it is necessary to deepen the understanding of the complex metabolic reprogramming within the TME (23).

## Immune cells

Tumor cells not only evade immune surveillance and defense but also create a hostile TME that perturb immune cell metabolism and corresponding anti-tumor immunity. Immune cells rely on diverse metabolic programs according to their cell type and function, and the immuno-metabolic interactions are critical for tumor development in the TME (24, 25). The mechanisms of how altered metabolism reprograms immune cell function are still being explored.

Tumor-associated macrophages (TAMs) exhibit a spectrum of metabolic and functional profiles in response to environmental stimuli, ranging from a pro-inflammatory and tumor-inhibiting M1-like state to a regulatory and tumor-promoting M2-like state (26). Metabolism governs macrophage polarization, activation, and antitumor immunity (27). Macrophages undergo a switch in their metabolic pathways that leads to differentiation into either M1 or M2 subtypes in the TME in response to cytokines produced by tumour cells (27). In this context, metabolic interventions may be effective in mediating anti-tumor effects that involve re-polarization of TAMs (26). M1-polarized macrophages always exhibit a glycolytic phenotype, while M2-polarized macrophages employ OXPHOS for bioenergetic synthesis with increased number of mitochondria and enhanced oxygen consumption rates (28). Thus, increased OXPHOS in TAMs may contribute to pro-tumorigenic effect, while targeting OXPHOS may be exploited to facilitate anti-tumorigenic functions (29).

Growing evidence has illustrated that distinct metabolic alterations are critical for effector function of T cell, including CD4+ and CD8+ T cells (30). Given that T cells are engaged in tumor development, it is necessary to elucidate the metabolic phenotype of T cell and its impact on anti-tumor efficacy and tumor progression. It has been demonstrated that ascites fluid collected from ovarian cancer patients could activate IRE1α-XBP1 ER stress in T cells to inhibit mitochondrial function and anti-tumor immunity. Inhibition of IRE1α-XBP1 activation can restore the OXPHOS and anti-tumor immunity against ovarian tumor (31). Increased L-arginine levels in tumor cells lead to metabolic switch from glycolytic phenotype to OXPHOS in activated T cells and enhance T cell survival and anti-tumor efficacy (32). Interleukin-10-Fc (IL-10/Fc) fusion protein has been identified to mediate proliferation and effector function of terminally exhausted CD8+ tumor infiltrating leukocytes *via* metabolic shift towards OXPHOS through the mitochondrial pyruvate carrier. This metabolic adaptation by enhancing OXPHOS can reverse terminally exhausted T cells and improve the efficacy to immunotherapy, indicating IL-10/Fc can synergize with other cancer immunotherapies for better clinical outcome (33). Based on these findings, OXPHOS is critical for effector function of T cells.

T regulatory cells (Tregs), an immunosuppressive subset of CD4+ T cells, maintain immunological homeostasis by enhancing self-tolerance and inhibiting autoimmune responses. Transcription factor Foxp3 is specifically expressed in Treg cells, and its expression is critical for differentiation and suppressive function of Tregs (34). Precious studies have reported that Tregs require OXPHOS for maintaining their suppressive capacity (35, 36). Tregs lacking Lkb1 have impaired mitochondria, reduced OXPHOS, and altered metabolic pathways that impair survival and suppressive capacity of Tregs (37). Foxp3 mediates metabolic rewiring of T cells by reducing Myc-mediated glycolysis and elevating OXPHOS. Thus, impairing electron transport chain complex I of Tregs could inhibit suppressive function of Tregs in tumors (38). Further studies are needed to explore the interaction between OXPHOS of Tregs and its function in ovarian cancer.

Therefore, the dysregulated oxidative energetics of tumor cells represent a metabolic vulnerability that could be exploited to enhance anti-tumor immunity. Metformin alone brings limited therapeutic benefit in highly aggressive tumors, whereas combination of metformin with PD-1 blockade improves anti-tumor effect of T cells (39). Metformin also mediates CD8+ tumor-infiltrating leukocyte proliferation and cytokine release, leading to an IFN-γ-dependent reprogramming of TME (40). Combination of radiotherapy and OXPHOS inhibitors can overcome PD-1 resistance and improve anti-tumor immunity (41). Impairing respiratory complex I can suppress immune checkpoints in multiple cancer models, uncovering a non-canonical role of electron transport chain inhibitors in regulating immune checkpoints to improve the anti-tumor efficacy (42).

## Adipocytes

Adipocytes are one of the main stromal cell types in multiple tissues, and thereby regarded as a key player in the TME. The adipocyte-tumor interaction results in metabolic and functional alteration within these cells, thereby promoting tumor development (43, 44). More importantly, ovarian tumors exhibit a tendency to metastasize and colonize to the omentum, a site that contains a large number of adipocytes (43, 44). Adipocytes store triglycerides and have been reported to transfer lipid directly to ovarian tumor cells in adipocyte-ovarian tumor cell co-culture, resulting in an increase in mitochondrial respiration to block ovarian cancer development (43–45). Moreover, increased mitochondrial respiration has been identified as a consequence of lipid transfer (36). The capabilities of ovarian tumor cells to adapt and colonize lipid-rich TME is essential for tumor development. Therefore, targeting metabolic interaction between adipocyte and tumor cells provides an opportunity for blocking ovarian cancer development. Dihydropyrimidinase-like 4 (DPYSL4), a member of the collapsin response mediator protein family, is involved in ovarian tumor development. DPYSL4 participates in the regulation of respiratory complexes I, III, and IV factors for supercomplex assembly to mediate OXPHOS. In preadipocytes, DPYSL4 overexpression can increase ATP production and oxygen consumption. DPYSL4 improves intracellular energy metabolism by localizing with mitochondrial super-complexes and regulating steps of the TCA cycle (46). Therefore, exploring key regulators of OXPHOS and metabolic adaptation in adipocytes may provide promising therapeutic targets for the treatment of ovarian tumors.

## Cancer-associated fibroblasts

Cancer-associated fibroblasts (CAFs) are an essential component of the TME and exhibit diverse functions to regulate tumor growth and metastasis. As such, CAFs are regarded as a promising target for optimizing therapeutic strategies against ovarian cancer. Numerous evidence has proposed that CAFs function as main regulators in shaping tumor metabolism especially through the dysregulation of several metabolic pathways. Thus, it is essential to uncover the mechanism of the CAFs-mediated metabolic shift to character metabolic vulnerabilities of ovarian tumors. ITGB2 promoted glycolysis through PI3K/AKT/mTOR pathways in CAFs and secreted lactate to promote OSCC proliferation by enhancing OXPHOS capacities. Using metformin to target the respiratory complex I could effectively inhibit the pro-proliferative effects of ITGB2-expressing CAFs. Lactate from ITGB2-expressing CAFs was absorbed and metabolized in oral squamous cell carcinoma to generate NADH to fuel tumor proliferation. Targeting respiratory complex I effectively inhibited the pro-proliferative effects of ITGB2 expressing CAFs, further supporting lactate oxidation in oral squamous cell carcinoma (47).

## Clinical implications for OXPHOS inhibition

The underlying mechanism of resistance to mitochondrial metabolic targeting agents is complex and dynamic. For instance, it has been demonstrated that BRCA1 deficiency upregulates N-nicotinamide methyltransferase (NNMT), which mediates metabolic reprogramming and sensitizes ovarian tumor cells to mitochondrial metabolic targeting agents. Mechanistically, BRCA1 depletion leads to metabolic adaptation of ovarian tumor cells by reducing mitochondrial respiration and ATP production (48). Loss of hexokinase 1 (HK1), a well-characterized enzyme engaged in glycolysis, can sensitize ovarian cancer to high-dose metformin (49). Hexokinase 2 (HK2) depletion suppresses glycolysis and enhances OXPHOS, which further sensitizes tumor cells to metformin. The combination of HK2 silencing and metformin synergistically induces cell death and suppress tumor growth (50). Collectively, synergistic inhibitors targeting mitochondrial respiration and specific metabolic vulnerabilities, such as glycolysis, open new avenues for anti-tumor strategies. Combination treatment of OXPHOS inhibitors with other chemotherapeutic agents and specific targeted therapies such as Src and EGFR inhibitors, may be potential therapeutic strategies (7). The mechanisms of resistance to mitochondrial metabolic targeting agents still require further investigations.

Exploring promising biomarkers for predicting therapeutic response to mitochondrial metabolic targeting agents is essential for precision treatment of ovarian cancer patients. Hig expression of PGC1α and β have been identified as biomarkers to select ovarian cancer patients that are more likely to benefit from metformin monotherapy. (19). Metformin monotherapy is also correlated with mitochondrial glycerol-3-phosphate dehydrogenase (MGPDH) downregulation and OXPHOS inhibition in tumor cells characterized by high MGPDH expression are more sensitive to metformin (51). It is necessary to identify molecular biomarkers to stratify patients that would benefit most from the treatment of OXPHOS inhibitors.

Targeting components of OXPHOS could open new avenues for cancer management. NDUFS1 is a nuclear encoded subunit of respiratory complex I. Xenografts established by CRISPR-Cas9 from NDUFS1−/− cells exhibit inhibited growth rates compared with control groups, making NDUFS1 a suitable target for therapeutic intervention. IACS-10759 inhibits OXPHOS by binding to mitochondrial respiratory complex 1 adjacent to the entrance of the ubiquinone channel to impair ubiquinone function, and is currently in phase I clinical development. Targeting OXPHOS with IACS-10759 inhibits growth of multiple tumor models with high antitumor efficacy (11).

## Conclusion

Numerous studies have revealed that OXPHOS is upregulated in some tumors, potentially rendering these tumors more sensitive to OXPHOS inhibition. Targeting OXPHOS has become a great potential option for anti-tumor treatment, and there are multiple studies indicating that OXPHOS inhibition is effective in some specific cancer types. However, specifically targeting one subpopulation may eventually fail and lead to a drug-resistant tumor. Inhibiting OXPHOS may result in selecting highly aggressive glycolytic subpopulations. Therefore, it is necessary to explore synergy between OXPHOS inhibitors and drugs blocking glycolysis. OXPHOS inhibition has been tested effectively in a series of tumor types. Identification of specific cancer types and/or molecular characteristics likely to respond to OXPHOS inhibition is required to enable stratification of patients most likely to benefit from this approach. Finally, combinations with other therapies should be further explored to improve the effect of anti-OXPHOS therapy.

## Author contributions

YW: Conceptualization and Writing of the first draft. XZ, ZW, HC: Review and Editing. WS: Conceptualization and Review. All authors contributed to the article and approved the submitted version.

## Conflict of interest

The authors declare that the research was conducted in the absence of any commercial or financial relationships that could be construed as a potential conflict of interest.

## Publisher’s note

All claims expressed in this article are solely those of the authors and do not necessarily represent those of their affiliated organizations, or those of the publisher, the editors and the reviewers. Any product that may be evaluated in this article, or claim that may be made by its manufacturer, is not guaranteed or endorsed by the publisher.
